# Direct-acting antiviral therapy is associated with a reduced risk of selected immune-mediated inflammatory diseases in chronic hepatitis C infection: A real-world cohort study

**DOI:** 10.1371/journal.pone.0351973

**Published:** 2026-06-25

**Authors:** Yeong-Jang Lin, Chia-Chih Kuo, Chih-Cheng Lai, Hung-An Chen, Chao-Yu Chen

**Affiliations:** 1 Division of Allergy, Immunology, and Rheumatology, Department of Internal Medicine, Chi Mei Medical Center, Tainan, Taiwan; 2 Center of General Education, Chia Nan University of Pharmacy and Science, Tainan, Taiwan; 3 Department of Internal Medicine, Chi Mei Medical Center, Tainan, Taiwan; 4 Department of Intensive Care Medicine, Chi Mei Medical Center, Tainan, Taiwan; University of Campania Luigi Vanvitelli: Universita degli Studi della Campania Luigi Vanvitelli, ITALY

## Abstract

**Background:**

Chronic hepatitis C virus (HCV) infection is associated with immune dysregulation and an increased risk of immune-mediated inflammatory diseases (IMIDs). While direct-acting antiviral (DAA) therapy achieves high rates of sustained virologic response, its long-term effects on the risk of IMIDs remain incompletely understood.

**Methods:**

We conducted a retrospective cohort study using data from the TriNetX global research network (2015–2024) to evaluate the association between DAA therapy and the risk of IMIDs among adults with chronic HCV infection. Patients were categorized into DAA-treated and untreated cohorts. Propensity score matching (1:1) was applied to balance baseline characteristics between groups. Cox proportional hazards models were used to estimate hazard ratios (HRs) and 95% confidence intervals (CIs). Sensitivity analyses, along with predefined positive and negative control outcomes, were performed to assess robustness. Subgroup analyses were conducted to assess potential effect modifiers.

**Results:**

After matching, 35,266 patients were included in each cohort. DAA therapy was associated with a significantly reduced risk of several IMIDs, including rheumatoid arthritis (HR 0.83, 95% CI 0.71–0.97), autoimmune hepatitis (HR 0.55, 95% CI 0.31–0.96), and immune thrombocytopenic purpura (HR 0.64, 95% CI 0.44–0.93). These associations were consistent across multiple predefined time-at-risk windows. Subgroup analyses revealed that the protective associations were more pronounced among females and middle-aged individuals (41–64 years).

**Conclusions:**

DAA therapy in patients with chronic HCV infection is associated with a reduced risk of specific IMIDs, suggesting potential systemic immunologic benefits beyond hepatic outcomes.

## Introduction

Hepatitis C virus (HCV) infection affects millions of people worldwide and is increasingly recognized as a systemic disease with extensive extrahepatic involvement, contributing substantially to overall morbidity and mortality [1]. Approximately 70% of patients with chronic HCV infection develop at least one extrahepatic manifestation, including mixed cryoglobulinemia, non-Hodgkin lymphoma, cardiovascular disease, renal impairment, insulin resistance, type 2 diabetes mellitus, and neuropsychiatric disorders [1]. In addition, HCV has been implicated in a broad spectrum of immune-mediated inflammatory diseases (IMIDs), such as Sjögren’s syndrome, rheumatoid arthritis, autoimmune thyroid disease, systemic lupus erythematosus, antiphospholipid syndrome, and autoimmune hepatitis [1–4].

Before the advent of direct-acting antivirals (DAAs), interferon-based regimens were the mainstay of HCV treatment [5]. However, the potent immunostimulatory effects of interferon often triggered or exacerbated IMIDs, limiting its use in patients with underlying immune dysregulation [6,7]. In contrast, DAA therapy—established as the standard of care since 2015—achieves sustained virologic response (SVR) rates exceeding 95% through direct viral inhibition, without the immunostimulatory effects associated with interferon [5].

Among HCV-associated IMIDs, mixed cryoglobulinemia represents one of the most extensively characterized conditions. Circulating cryoglobulins are detectable in up to 60% of patients with chronic HCV infection, and symptomatic cryoglobulinemic vasculitis develops in approximately 15%, most commonly involving the skin, kidneys, and peripheral nerves [8]. The introduction of DAA therapy has substantially improved clinical outcomes in this population. For instance, a prospective multicenter Italian cohort study demonstrated that DAA-induced SVR was associated with clinical improvement in more than 70% of patients with cryoglobulinemic vasculitis, accompanied by reductions in cryoglobulin levels and restoration of complement C4, suggesting that viral eradication may partially restore immune homeostasis [9].

Despite the well-documented cardiovascular and renal benefits of DAA therapy [10,11], robust large-scale evidence regarding its effect on the risk of incident IMIDs remains limited. This knowledge gap is particularly critical because the incidence of individual IMIDs is relatively low, thereby necessitating large, diverse datasets to achieve sufficient statistical power. Moreover, it remains uncertain whether the potential immunomodulatory effects are modified by age, sex, or race—demographic factors known to influence immune function and disease susceptibility. Sex-based differences are increasingly recognized as important determinants of immune regulation and HCV disease progression [12–14]. Females generally exhibit stronger innate and adaptive immune responses compared to males [12], which may contribute to both higher rates of spontaneous viral clearance and increased susceptibility to various IMIDs [13,14]. Such sex-related differences may therefore influence the long-term immunologic consequences of DAA therapy and warrant specific investigation.

We hypothesized that DAA therapy would be associated with a reduced risk of incident IMIDs through attenuation of chronic immune activation associated with persistent HCV infection. Given known biological variations in immune responses, we explored whether these associations differed according to demographic characteristics such as age and sex.

To address these unmet needs, we utilized the TriNetX global research network to evaluate the association between DAA therapy and the risk of incident IMIDs in a large, diverse, real-world cohort of patients with chronic HCV infection.

## Methods

### Study design and data source

This retrospective cohort study was conducted using data from TriNetX, a global federated health research network comprising de-identified electronic health records from over 300 million patients across more than 120 healthcare organizations worldwide [15]. TriNetX captures comprehensive clinical data, including demographics, diagnoses (International Classification of Diseases, Tenth Revision, Clinical Modification, ICD-10-CM), procedures, medications, laboratory results, and healthcare utilization.

This study used de-identified electronic health record data from the TriNetX platform. The use of such anonymized data was determined by the Western Institutional Review Board to be exempt from Institutional Review Board approval, and the requirement for informed consent was waived, as the study does not constitute human subjects research under applicable federal regulations.

The authors did not have access to information that could identify individual participants during or after data collection.

Data analysis was conducted on May 23, 2025, and the study period spanned from January 1, 2015, to December 31, 2024.

### Study population

Adult patients (aged ≥ 18 years) with active chronic HCV infection were identified using ICD-10-CM code B18.2 together with a documented positive HCV RNA test result. This dual-criteria approach was employed to exclude individuals with spontaneously resolved or previously treated infections.

Patients were excluded based on the following criteria: (1) a diagnosis of hepatitis B virus (HBV) or human immunodeficiency virus (HIV) infection before the index date, to eliminate confounding from alternative sources of immune dysregulation; (2) receipt of interferon or ribavirin during the study period, to isolate the effects of DAAs from other immunomodulatory agents; (3) a documented diagnosis of any study-defined IMID before the index date, to ensure that all outcomes represented incident cases; or (4) death documented before the index date.

### Exposure definition

Patients were categorized into two mutually exclusive cohorts according to their exposure to DAA therapy during the study period:

- DAA-treated cohort: This cohort included patients who initiated treatment with specific high-potency, interferon-free DAA regimens, including sofosbuvir/velpatasvir, sofosbuvir/ledipasvir, glecaprevir/pibrentasvir, or sofosbuvir/velpatasvir/voxilaprevir. The index date was defined as the date of the first prescription of any of these agents.
- Untreated cohort: This cohort consisted of patients with no documented exposure to any DAA agents—including both the study-specified regimens and earlier generations of DAAs—at baseline or throughout the entire follow-up period. The index date was defined as the date of the first documented positive HCV RNA test.

### Outcomes

A total of 13 IMIDs were pre-specified as primary outcomes based on prior evidence linking these conditions to chronic HCV infection. Patients with a diagnosis of any of these conditions before the index date were excluded to ensure the identification of incident cases. IMIDs were ascertained using ICD-10-CM diagnostic codes (S1 Table).

These outcomes encompassed both systemic and organ-specific IMIDs:

- Systemic IMIDs: rheumatoid arthritis, systemic lupus erythematosus, Sjögren’s syndrome, systemic sclerosis, dermatopolymyositis, sarcoidosis, systemic vasculitis, and antiphospholipid syndrome
- Organ-specific IMIDs: autoimmune hepatitis, autoimmune thyroiditis, immune thrombocytopenic purpura, cutaneous vasculitis, and psoriasis

To evaluate internal validity and assess residual confounding, predefined positive and negative control outcomes were incorporated:

- Positive controls (expected to improve following HCV eradication): liver cirrhosis, hepatocellular carcinoma, cryoglobulinemia, and type 2 diabetes mellitus
- Negative controls (conditions without established biological or clinical links to HCV infection or DAA therapy): osteoarthritis, migraine, and acute appendicitis

To mitigate reverse causation, early-detection bias, and potential immortal time bias, outcomes occurring within the first 180 days after the index date were excluded. Follow-up began on day 181 post-index and continued until the occurrence of the outcome, death, loss to follow-up, or the end of the study.

### Covariates and propensity score matching

To minimize confounding, 1:1 propensity score matching was conducted within the TriNetX platform using greedy nearest-neighbor matching with a fixed caliper of 0.1. Covariates were selected based on their potential as confounders or established risk factors for IMIDs. Baseline covariates were assessed during the 12 months prior to the index date. All covariates were identified using ICD-10-CM codes unless otherwise specified. The following covariates were included:

- Demographics: age at index, sex, race, and adverse socioeconomic status (ICD-10-CM codes indicating problems related to housing and economic circumstances, employment and unemployment problems, education and literacy problems, and occupational exposure to risk factors)
- Lifestyle-related proxies: tobacco use and nicotine dependence (for smoking), and alcoholic liver disease (for alcohol consumption)
- Comorbidities: hypertension, type 2 diabetes mellitus, hyperlipidemia, chronic kidney disease, asthma, depression, sleep disorder, psychoactive substance use, vitamin D deficiency, liver cirrhosis, hepatic fibrosis, and neoplasms
- Body mass index

### Statistical analyses

Baseline characteristics were compared using Student’s *t*-tests for continuous variables and Pearson’s chi-squared tests for categorical variables. Covariate balance after matching was evaluated using standardized mean differences, with values <0.1 indicating adequate balance.

Incidence rates were calculated as events per 1,000 person-years. Cox proportional hazards models were used to estimate hazard ratios (HRs) and 95% confidence intervals (CIs). The proportional hazards assumption was tested using the generalized Schoenfeld approach built in the TriNetX platform. Kaplan-Meier survival curves were generated to visualize time-to-event distributions.

Sensitivity analyses were performed by varying the post-index exclusion window (60, 180, and 365 days) to assess the robustness of the findings. Subgroup analyses were conducted to evaluate potential effect modification by age, sex, and race.

All statistical tests were two-sided, and a P value < 0.05 was considered statistically significant.

A complete list of ICD-10-CM codes used to define exposures, outcomes, covariates, and exclusion criteria is provided in S1 Table.

### Time zero definition and potential immortal time bias

Because initiation of DAA therapy may occur at variable intervals following HCV diagnosis, particular attention was paid to defining time zero and addressing the risk of immortal time bias. Cohort entry (time zero) was therefore specified separately for each exposure group. For the DAA-treated cohort, the index date was defined as the date of the first DAA prescription. For the untreated cohort, the index date corresponded to the date of the first documented positive HCV RNA test during the study period, representing the earliest confirmed evidence of active HCV infection in the electronic health record.

Because patients in the treated cohort must survive and remain event-free until initiation of DAA therapy, immortal time between HCV diagnosis and treatment initiation may be introduced. To mitigate this bias, outcomes occurring within a predefined post-index exclusion window were excluded from both cohorts. The primary exclusion window was set at 180 days, with additional sensitivity analyses applying alternative windows of 60 and 365 days to evaluate the robustness of the results under varying assumptions regarding time at risk. Although these methodological approaches reduce early-event and time-related bias, residual bias cannot be entirely excluded. Consequently, the findings should be interpreted as associative rather than causal.

## Results

### Study population

**Fig 1:**
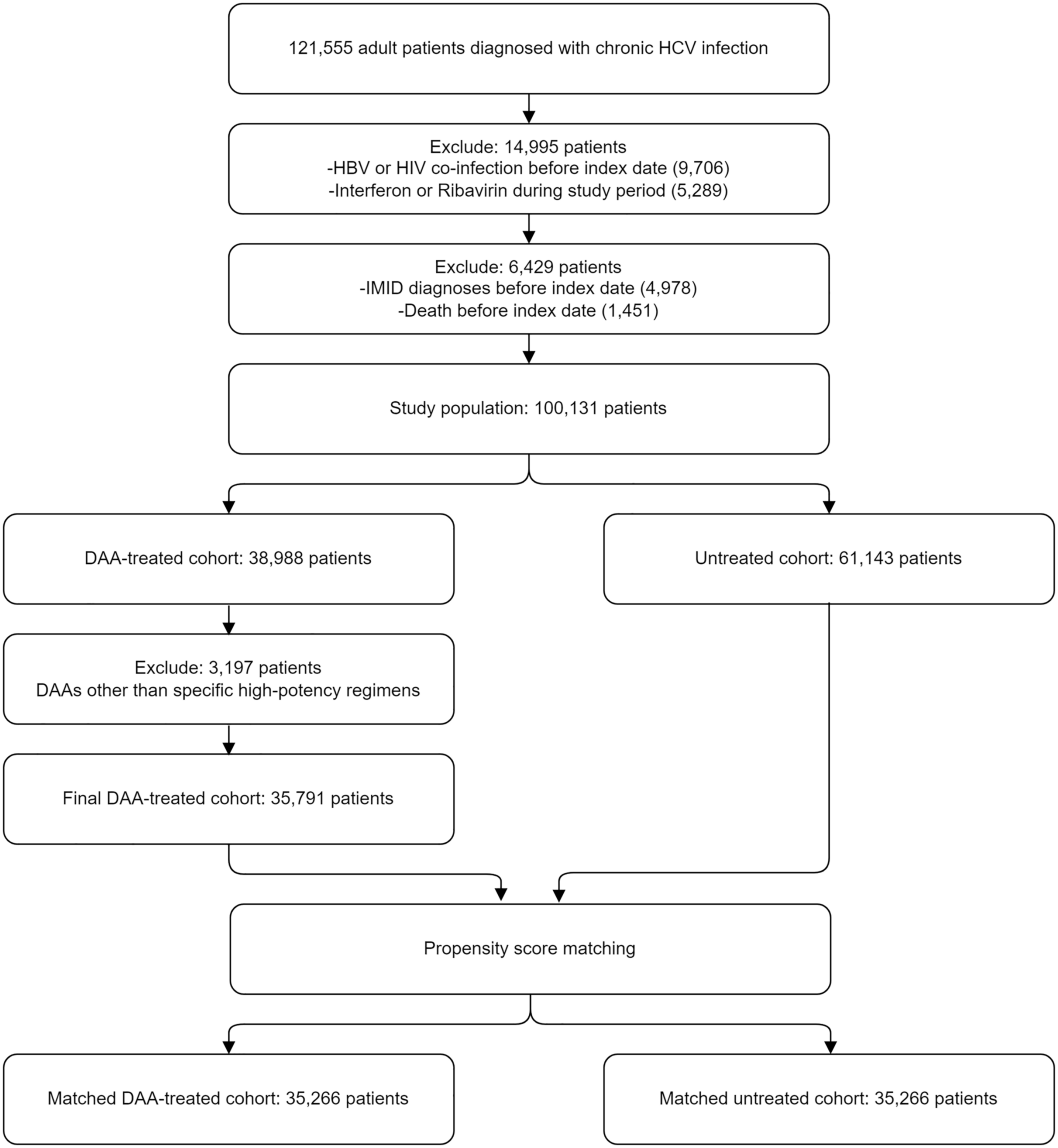
Flowchart of cohort selection. The figure depicts the inclusion and exclusion criteria used to identify eligible patients with chronic HCV infection, and the final study populations in the DAA-treated and untreated groups following 1:1 propensity score matching. Abbreviations: HCV, hepatitis C virus; HBV, hepatitis B virus; HIV, human immunodeficiency virus; DAA, direct-acting antiviral.

Fig 1 illustrates the patient selection process. A total of 121,555 adults with chronic active HCV infection were identified during the study period. After exclusion of 14,995 individuals with HBV or HIV co-infection or interferon-based therapy, and 6,429 patients with pre-existing IMIDs or baseline mortality, an analytical cohort of 100,131 eligible patients remained.

Patients were subsequently stratified according to DAA exposure status. The initial DAA-treated cohort included 38,988 patients, of whom 3,197 were excluded for not meeting the predefined regimen criteria, yielding a final treated cohort of 35,791 patients. The untreated cohort consisted of 61,143 DAA-naïve patients.

After 1:1 propensity score matching, two well-balanced cohorts of 35,266 patients each were established for comparative analyses.

### Baseline characteristics

Table 1 presents baseline characteristics of the DAA-treated and untreated cohorts before and after propensity score matching. After matching, all covariates achieved excellent balance, with standardized mean differences < 0.1 across demographic, socioeconomic, lifestyle, and comorbidity variables. In the matched population, the mean age of the DAA-treated cohort was 51.4 years (standard deviation, 13.8), 59.9% of patients were male, and 64.4% were White.

**Table 1:** Baseline characteristics of DAA-treated and untreated patients before and after propensity score matching.

Variables	Before matching, No. (%)			After matching, No. (%)		
	DAA-treated	Untreated	SMD	DAA-treated	Untreated	SMD
	(n = 35,791)	(n = 61,143)		(n = 35,266)	(n = 35,266)	
Age at index, mean ± SD, years	51.5 ± 13.8	48.7 ± 14.5	0.196	51.4 ± 13.8	51.4 ± 14.1	<0.001
Sex						
Male	21,430 (59.9)	36,651 (59.9)	0.001	21,115 (59.9)	21,268 (60.3)	0.009
Female	13,559 (37.9)	23,119 (37.8)	0.001	13,365 (37.9)	13,196 (37.4)	0.010
Race						
White	23,023 (64.3)	40,686 (66.5)	0.047	22,706 (64.4)	22,442 (63.6)	0.016
Black or African American	8,385 (23.4)	12,430 (20.3)	0.075	8,234 (23.3)	8,227 (23.3)	<0.001
Asian	474 (1.3)	733 (1.2)	0.011	467 (1.3)	485 (1.4)	0.004
Socioeconomic status						
Housing/Economic circumstances	762 (2.1)	2614 (4.3)	0.122	761 (2.2)	716 (2.0)	0.009
Employment/Unemployment problems	116 (0.3)	432 (0.7)	0.053	116 (0.3)	111 (0.3)	0.003
Education and literacy problems	26 (0.1)	64 (0.1)	0.011	26 (0.1)	24 (0.1)	0.002
Occupational exposure to risk factors	21 (0.1)	18 (0.0)	0.014	21 (0.1)	18 (0.1)	0.004
Lifestyle						
Tobacco use (smoking)	2,891 (8.1)	4,545 (7.4)	0.024	2,842 (8.1)	2,951 (8.4)	0.011
Nicotine dependence (smoking)	10,740 (30.0)	18,336 (30.0)	<0.001	10,603 (30.1)	11,165 (31.7)	0.035
Alcoholic liver disease (alcohol use)	1,582 (4.4)	2,633 (4.3)	0.006	1,557 (4.4)	1,565 (4.4)	0.001
Comorbidities						
Hypertension	11,988 (33.5)	14,926 (24.4)	0.201	11,635 (33.0)	11,604 (32.9)	0.002
Type 2 diabetes mellitus	4,758 (13.3)	6,489 (10.6)	0.083	4,618 (13.1)	4,715 (13.4)	0.008
Hyperlipidemia	3,926 (11.0)	4,186 (6.8)	0.145	3,732 (10.6)	3,559 (10.1)	0.016
Chronic kidney disease	2,523 (7.0)	3,316 (5.4)	0.067	2,437 (6.9)	2,488 (7.1)	0.006
Asthma	2,260 (6.3)	3,086 (5.0)	0.055	2,193 (6.2)	2,209 (6.3)	0.002
Depression	5,226 (14.6)	7,366 (12.0)	0.075	5,093 (14.4)	5,209 (14.8)	0.009
Sleep disorder	3,326 (9.3)	3,666 (6.0)	0.124	3,156 (8.9)	3,033 (8.6)	0.012
Psychoactive substance use	16,170 (45.2)	26,997 (44.2)	0.021	15,951 (45.2)	16,755 (47.5)	0.046
Vitamin D deficiency	2,045 (5.7)	1,810 (3.0)	0.135	1,889 (5.4)	1,658 (4.7)	0.030
Liver cirrhosis	5,593 (15.6)	5,381 (8.8)	0.210	5,236 (14.8)	4,705 (13.3)	0.043
Hepatic fibrosis	2,216 (6.2)	1,636 (2.7)	0.171	2,005 (5.7)	1,561 (4.4)	0.057
Neoplasms	5,115 (14.3)	5,630 (9.2)	0.158	4,885 (13.9)	4,694 (13.3)	0.016
Body mass index, mean ± SD, kg/m^2^	28.1 ± 6.4	27.3 ± 6.3	0.131	28.1 ± 6.4	27.5 ± 6.4	0.094

Abbreviations: DAA, direct-acting antiviral; SD, standard deviation; SMD, standardized mean difference.

### Incidence of immune-mediated inflammatory diseases

Table 2 summarizes the incidence rates and HRs for individual IMIDs and control outcomes. Testing of the proportional hazards assumption using the generalized Schoenfeld approach revealed no significant violations for any primary outcome (all P > 0.05), supporting the validity of the Cox proportional hazards models. Among the 13 IMIDs evaluated, DAA therapy was associated with significantly lower risks of rheumatoid arthritis (HR 0.83, 95% CI 0.71–0.97), autoimmune hepatitis (HR 0.55, 95% CI 0.31–0.96), and immune thrombocytopenic purpura (HR 0.64, 95% CI 0.44–0.93).

**Table 2:** Incidence rates and hazard ratios of immune-mediated inflammatory diseases and control outcomes in the DAA-treated and untreated cohorts.

Outcomes	DAA-treated		Untreated			
	Events	Incidence rate	Events	Incidence rate	Hazard ratio	P-value
		/1,000 PY (95% CI)		/1,000 PY (95% CI)	(95% CI)	
**Systemic IMID**						
Rheumatoid arthritis	294	2.17 (1.94–2.43)	368	2.62 (2.37–2.91)	**0.83 (0.71–0.97)**	**0.018**
Systemic lupus erythematosus	47	0.35 (0.26–0.46)	61	0.43 (0.34–0.56)	0.81 (0.55–1.19)	0.279
Sjögren’s syndrome	64	0.47 (0.37–0.60)	52	0.37 (0.28–0.49)	1.29 (0.90–1.86)	0.169
Systemic sclerosis	12	0.09 (0.05–0.16)	≤ 10†	–	1.54 (0.63–3.76)	0.343
Dermatopolymyositis	≤ 10†	–	≤ 10†	–	0.65 (0.24–1.79)	0.399
Sarcoidosis	44	0.32 (0.24–0.44)	40	0.28 (0.21–0.39)	1.13 (0.74–1.74)	0.571
Systemic vasculitis	71	0.52 (0.41–0.66)	100	0.71 (0.59–0.87)	0.74 (0.55–1.01)	0.054
Antiphospholipid syndrome	22	0.16 (0.11–0.25)	20	0.14 (0.09–0.22)	1.14 (0.62–2.09)	0.674
**Organ-specific IMID**						
Autoimmune hepatitis	19	0.14 (0.09–0.22)	35	0.25 (0.18–0.35)	**0.55 (0.31–0.96)**	**0.033**
Autoimmune thyroiditis	83	0.61 (0.49–0.76)	72	0.51 (0.41–0.65)	1.20 (0.88–1.65)	0.258
Immune thrombocytopenic purpura	44	0.32 (0.24–0.44)	71	0.51 (0.40–0.64)	**0.64 (0.44–0.93)**	**0.019**
Cutaneous vasculitis	40	0.29 (0.22–0.40)	54	0.38 (0.29–0.50)	0.77 (0.51–1.15)	0.198
Psoriasis	242	1.79 (1.57–2.03)	222	1.58 (1.39–1.80)	1.14 (0.95–1.37)	0.166
**Positive control outcome**						
Liver cirrhosis	1,907	18.87 (18.04–19.74)	2,896	27.20 (26.22–28.20)	**0.72 (0.68–0.76)**	**<0.001**
Hepatocellular carcinoma	705	5.35 (4.97–5.76)	834	6.28 (5.87–6.72)	**0.88 (0.80–0.97)**	**0.013**
Cryoglobulinemia	46	0.34 (0.26–0.46)	71	0.51 (0.40–0.64)	**0.66 (0.45–0.95)**	**0.026**
Type 2 diabetes mellitus	1,542	13.68 (13.02–14.38)	1,983	17.41 (16.66–18.19)	**0.79 (0.74–0.85)**	**<0.001**
**Negative control outcome**						
Osteoarthritis	6,650	49.01 (47.85–50.21)	6,787	48.26 (47.13–49.43)	1.01 (0.98–1.04)	0.579
Migraine	1,424	10.50 (9.96–11.06)	1,369	9.74 (9.23–10.27)	1.06 (0.99–1.14)	0.116
Acute appendicitis	84	0.62 (0.50–0.77)	93	0.66 (0.54–0.81)	0.94 (0.70–1.26)	0.673

Bold font indicates statistically significant results (*P* < 0.05).

†To protect patient privacy, numbers are rounded up to 10.

Abbreviations: DAA, direct-acting antiviral; PY, person-year; CI, confidence interval; IMID, immune-mediated inflammatory disease.

**Fig 2:**
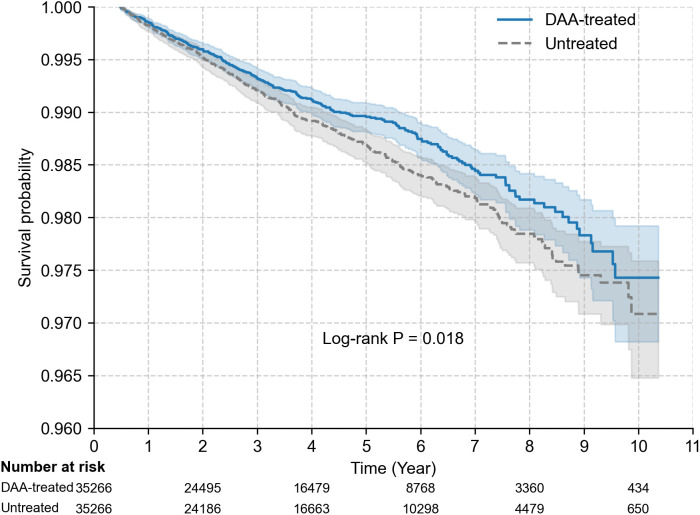
Kaplan-Meier survival curves for rheumatoid arthritis. The curves illustrate the cumulative probability of remaining free from rheumatoid arthritis over time in the DAA-treated and untreated cohorts. Shaded areas represent 95% confidence intervals. Log-rank P = 0.018. Abbreviations: DAA, direct-acting antiviral.

Kaplan-Meier analysis demonstrated a significantly higher probability of remaining free from rheumatoid arthritis among patients receiving DAA therapy (log-rank P = 0.018; Fig 2). Kaplan-Meier curves were not generated for autoimmune hepatitis or immune thrombocytopenic purpura owing to the limited number of events, which could yield unstable estimates.

No statistically significant associations were observed for other IMIDs, such as systemic lupus erythematosus (HR 0.81, 95% CI 0.55–1.19), systemic vasculitis (HR 0.74, 95% CI 0.55–1.01), and cutaneous vasculitis (HR 0.77, 95% CI 0.51–1.15). Notably, Sjögren’s syndrome (HR 1.29, 95% CI 0.90–1.86), sarcoidosis (HR 1.13, 95% CI 0.74–1.74), autoimmune thyroiditis (HR 1.20, 95% CI 0.88–1.65), and psoriasis (HR 1.14, 95% CI 0.95–1.37) exhibited numerically higher, but statistically non-significant, risks in the DAA-treated cohort, with CIs crossing unity.

DAA therapy was also associated with significantly reduced risk of all positive control outcomes, including liver cirrhosis (HR 0.72, 95% CI 0.68–0.76), hepatocellular carcinoma (HR 0.88, 95% CI 0.80–0.97), cryoglobulinemia (HR 0.66, 95% CI 0.45–0.95), and type 2 diabetes mellitus (HR 0.79, 95% CI 0.74–0.85). In contrast, no significant associations were observed for negative control outcomes, including osteoarthritis (HR 1.01, 95% CI 0.98–1.04), migraine (HR 1.06, 95% CI 0.99–1.14), and acute appendicitis (HR 0.94, 95% CI 0.70–1.26). Collectively, these findings support the internal validity of the study and the specificity of the observed associations.

### Sensitivity analyses

Sensitivity analyses using alternative post-index exclusion windows of 60 and 365 days yielded results consistent with the primary analysis (S2 Table). The HRs for rheumatoid arthritis and immune thrombocytopenic purpura remained stable across all risk-window definitions, ranging from 0.83 to 0.84 for rheumatoid arthritis and from 0.64 to 0.65 for immune thrombocytopenic purpura. The consistency of these findings across varying assumptions regarding time at risk underscores the robustness of the observed associations.

### Stratified analyses

**Fig 3:**
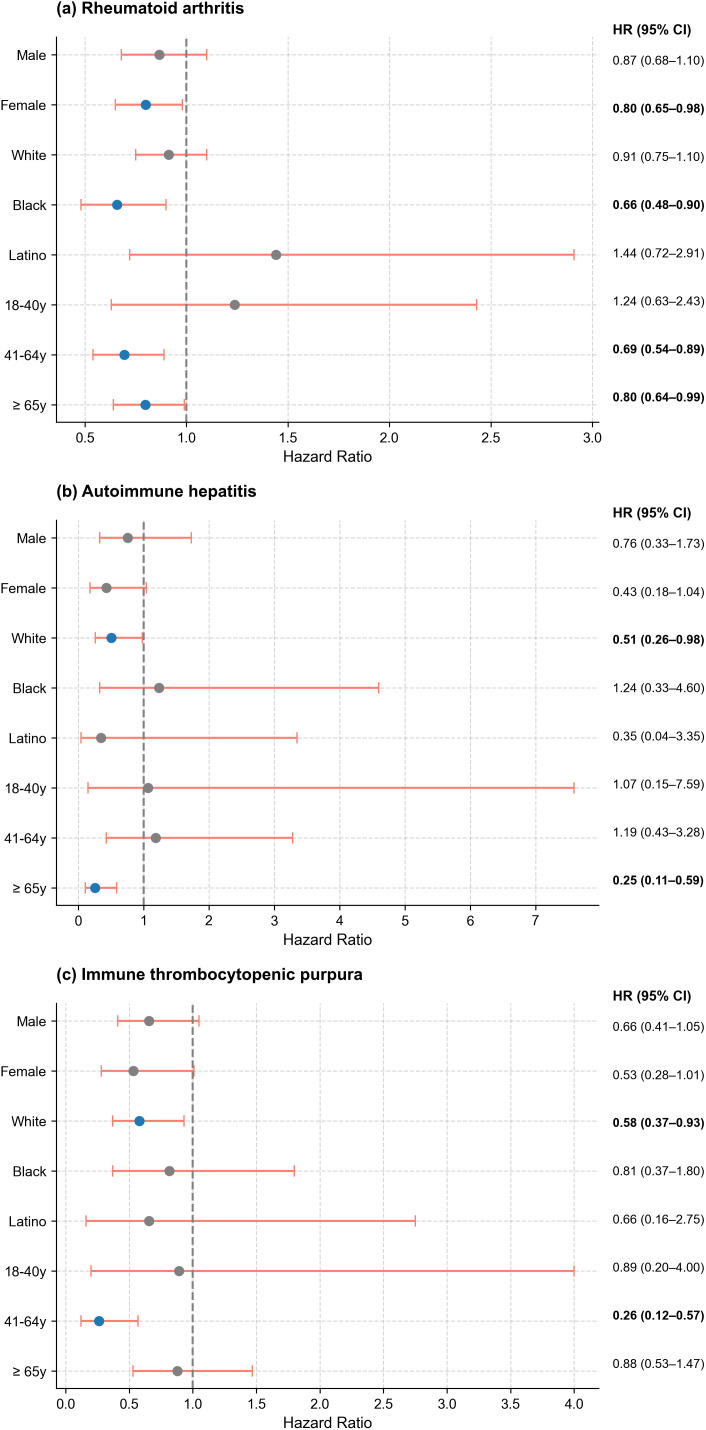
Stratified analysis of hazard ratios for selected immune-mediated inflammatory diseases. Forest plots display hazard ratios with 95% confidence intervals for rheumatoid arthritis (Panel a), autoimmune hepatitis (Panel b), and immune thrombocytopenic purpura (Panel c) across demographic subgroups (age, sex, race). Subgroup-specific estimates are provided in S3 Table. Abbreviations: HR, hazard ratio; CI, confidence interval.

Exploratory subgroup analyses suggested potential heterogeneity of associations across demographic strata. For rheumatoid arthritis (Fig 3a), statistically significant risk reductions were observed among females (HR 0.80, 95% CI 0.65–0.98), Black patients (HR 0.66, 95% CI 0.48–0.90), and individuals aged 41–64 years (HR 0.69, 95% CI 0.54–0.89) and ≥65 years (HR 0.80, 95% CI 0.64–0.99).

For autoimmune hepatitis (Fig 3b), significantly reduced risks were observed among White patients (HR 0.51, 95% CI 0.26–0.98) and those aged ≥65 years (HR 0.25, 95% CI 0.11–0.59). For immune thrombocytopenic purpura (Fig 3c), lower risks were observed among White patients (HR 0.58, 95% CI 0.37–0.93) and individuals aged 41–64 years (HR 0.26, 95% CI 0.12–0.57).

Detailed subgroup-specific event counts and corresponding effect estimates are provided in S3 Table.

## Discussion

In this large-scale, real-world cohort study, DAA therapy in patients with chronic HCV infection was associated with a modest but statistically significant reduction in the risk of several IMIDs. Specifically, the risks of rheumatoid arthritis, autoimmune hepatitis, and immune thrombocytopenic purpura were significantly lower in the DAA-treated cohort. These findings suggest that DAA therapy in patients with chronic HCV infection may confer systemic immunological benefits that extend beyond hepatic outcomes.

A biologically plausible explanation for the observed associations may involve attenuation of chronic systemic inflammation following DAA therapy. Persistent HCV infection promotes immune dysregulation through sustained antigenic stimulation, T helper 17 (Th17) cell expansion, and B-cell hyperactivation—mechanisms implicated in the pathogenesis of IMIDs [16,17]. Our findings are consistent with prior observations of clinical improvement in HCV-associated cryoglobulinemic vasculitis after achievement of SVR [9], supporting the hypothesis that reduction of chronic viral antigenic stimulation following DAA therapy may facilitate partial restoration of immune homeostasis.

Notably, the protective associations were not uniform across all IMIDs. For Sjögren’s syndrome, sarcoidosis, autoimmune thyroiditis, and psoriasis, we observed numerically higher but statistically non-significant HRs in the DAA-treated cohort. These results contrast with previous studies reporting increased risks of these conditions in the setting of chronic HCV infection [2,18,19] and may reflect diagnostic ascertainment bias. Patients receiving DAA therapy often undergo more frequent post-treatment surveillance and laboratory monitoring, which may increase the likelihood of detecting certain IMIDs. Importantly, the persistence of significant risk reductions for rheumatoid arthritis, autoimmune hepatitis, and immune thrombocytopenic purpura despite this potential bias strengthens the credibility of the observed protective associations.

Importantly, some immune-mediated manifestations in chronic HCV infection may clinically resemble classical autoimmune diseases, including HCV-related arthritis mimicking rheumatoid arthritis, lupus-like syndromes resembling systemic lupus erythematosus, and autoimmune hepatitis-like manifestations, potentially contributing to diagnostic overlap in electronic health record-based studies. In addition, outcome ascertainment in this study relied on ICD-10-CM diagnostic coding and therefore reflects clinically documented IMIDs in real-world practice rather than adjudicated autoimmune disease phenotypes. Accordingly, the associations observed in this study should not be interpreted as evidence of direct causality between HCV infection and idiopathic autoimmune diseases, but rather as associations involving clinically documented immune-mediated inflammatory conditions in routine electronic health record-based practice.

Subgroup analyses suggested that the protective effects of DAA therapy were more pronounced among females and middle-aged individuals. These differences may be attributable to immunogenetic factors and sex hormone-mediated modulation of immune responses. Females generally exhibit more robust humoral and cellular immune activity and a higher susceptibility to IMIDs, which may amplify the degree of immunologic recalibration following attenuation of chronic viral stimulation [12]. Sex-related differences in chronic liver disease progression and antiviral immune responses are increasingly recognized in HCV infection [14,20]. Estrogen signaling, differential cytokine profiles, and variations in innate and adaptive immune responses may contribute to sex-specific manifestations of both hepatic and extrahepatic disease [14,20]. Estrogen signaling has been shown to exert anti-inflammatory and anti-fibrotic effects through inhibition of hepatic stellate cell activation and modulation of immune responses [20]. In chronic liver disease, these biological differences are reflected by slower fibrosis progression in premenopausal women than in men or postmenopausal women [20]. Beyond the liver, similar immune regulatory differences may also contribute to a greater restoration of immune homeostasis following DAA therapy, potentially explaining the more pronounced associations observed among female patients in our cohort.

The immunological consequences of chronic viral infection and the immunologic effects of antiviral therapy have attracted increasing attention in recent years. Beyond HCV, other viruses—including Epstein-Barr virus (EBV) and severe acute respiratory syndrome coronavirus 2 (SARS-CoV-2)—have been implicated in the pathogenesis of IMIDs [21,22]. EBV has been strongly associated with systemic lupus erythematosus, multiple sclerosis, and rheumatoid arthritis [21], with prospective evidence suggesting that EBV seroconversion may precede the onset of multiple sclerosis [23]. However, because effective EBV eradication therapies are lacking, whether viral suppression reduces long-term IMID risk remains speculative. In contrast, emerging evidence suggests that recovery from SARS-CoV-2 infection does not uniformly reduce autoimmune risk, as large cohort studies have demonstrated persistently elevated IMID incidence long after clinical recovery [24]. Collectively, these observations highlight that attenuation of chronic viral antigenic stimulation does not invariably lead to resolution of immune-mediated pathology and that outcomes likely depend on virus-specific immunopathology, host susceptibility, and the nature of post-treatment immune reconstitution. Within this conceptual framework, our findings suggest an association between DAA therapy and reduced risk of selected IMIDs in chronic HCV infection.

### Strengths and limitations

This study has several notable strengths. We leveraged a large, multinational real-world cohort from the TriNetX global research network, enabling adequate statistical power to evaluate relatively uncommon IMIDs. Propensity score matching with comprehensive adjustment for demographic characteristics, socioeconomic proxies, comorbidities, and lifestyle-related variables yielded well-balanced comparison groups. The inclusion of both positive and negative control outcomes further supported the internal validity and specificity of the analytical approach. Importantly, treatment crossover was not permitted by design; individuals in the untreated cohort were required to have no documented exposure to any DAA agents throughout the entire observation period. This methodological feature minimizes bias from post-index treatment initiation in the comparator group—a limitation common to prior observational studies of DAA outcomes. By maintaining mutually exclusive exposure groups and applying multiple post-index exclusion windows, our study enhances the interpretability of long-term associations between DAA therapy and incident IMIDs.

Several limitations should be acknowledged. Selection bias is an inherent concern in retrospective observational studies. Initiation of DAA therapy was not randomized and may have been influenced by factors such as liver disease severity, comorbidity burden, access to healthcare, and provider or patient preferences, which could affect both treatment allocation and outcome detection. Although propensity score matching was used to balance measured baseline covariates, residual confounding and surveillance bias related to differential healthcare utilization and other unmeasured factors cannot be fully excluded.

In addition, time-related biases are an important consideration in observational treatment studies. The index date for the DAA-treated cohort was defined as treatment initiation, whereas the untreated cohort was indexed at the time of confirmed HCV RNA positivity, potentially introducing differences in disease duration. A key concern is immortal time bias, as patients in the DAA-treated cohort must survive and remain event-free until treatment initiation. To mitigate this concern, we applied a predefined 180-day post-index exclusion window, excluding outcomes occurring during this interval to reduce reverse causation and early-event bias. Sensitivity analyses using alternative lag periods of 60 and 365 days yielded consistent HRs for primary outcomes, supporting the robustness of the findings across varying assumptions of time at risk. Moreover, the validity of our findings is reinforced by the negative control outcomes—osteoarthritis, migraine, and acute appendicitis—which demonstrated HRs close to unity. If the observed protective associations were primarily driven by immortal time bias or healthy-user effects, similar reductions might also be expected for these unrelated outcomes. The absence of such patterns supports the specificity of the associations, although causal inference should still be interpreted cautiously.

Furthermore, SVR status was not systematically available in the TriNetX platform, precluding direct differentiation between DAA exposure and confirmed virologic cure. Accordingly, the observed associations should be interpreted in the context of DAA exposure rather than confirmed virologic eradication. Information regarding treatment duration, medication adherence, and treatment discontinuation was not consistently available across participating healthcare organizations and therefore could not be reliably analyzed. Future studies incorporating more detailed treatment-related and virologic data are warranted. Outcome misclassification is possible because diagnoses were identified using single ICD-10-CM codes, which may reduce specificity for some IMIDs. However, outcome definitions were applied uniformly across exposure groups, and any resulting misclassification is likely to be non-differential, potentially biasing estimates toward the null. In addition, metabolic dysfunction-associated steatotic liver disease (MASLD) may independently influence systemic inflammatory and immune responses, potentially acting as a confounder [25]. Studies have shown variations in MASLD prevalence and metabolic outcomes following DAA therapy [26,27]. While we addressed major metabolic components (e.g., body mass index, type 2 diabetes, and dyslipidemia) through propensity score matching, specific clinical indicators for MASLD, such as liver imaging, histology, or standardized metabolic phenotyping, were not consistently available within the TriNetX platform. Consequently, longitudinal changes in MASLD status before and after DAA therapy could not be reliably evaluated. Therefore, residual confounding related to the presence or severity of MASLD cannot be entirely excluded. Finally, given the evaluation of multiple IMIDs, the possibility of chance findings due to multiple comparisons cannot be entirely excluded.

## Conclusion

In conclusion, DAA therapy was associated with a significantly lower risk of incident rheumatoid arthritis, autoimmune hepatitis, and immune thrombocytopenic purpura among patients with chronic HCV infection. These findings suggest that DAA therapy may confer systemic immunologic benefits extending beyond hepatic outcomes. Future studies incorporating confirmed SVR status, detailed immunologic profiling, and healthcare utilization metrics are warranted to further clarify the long-term immunologic consequences of DAA therapy in chronic HCV infection.

## Supporting information

S1 Table ICD-10-CM code definitions. Comprehensive list of all ICD-10-CM codes used in the study, including definitions for immune-mediated inflammatory diseases, inclusion and exclusion criteria, covariates used for propensity score matching, and control outcomes. Abbreviations: HCV, hepatitis C virus; HBV, hepatitis B virus; HIV, human immunodeficiency virus.
(DOCX)

S2 Table Sensitivity analyses using alternative post-index exclusion windows. Hazard ratios for key immune-mediated inflammatory diseases (rheumatoid arthritis, autoimmune hepatitis, and immune thrombocytopenic purpura) under 60-day, 180-day (primary), and 365-day exclusion windows to assess robustness. Abbreviations: HR, hazard ratio; CI, confidence interval.
(DOCX)

S3 Table Stratified analysis of immune-mediated inflammatory disease risk by demographic subgroups. Hazard ratios and 95% confidence intervals for rheumatoid arthritis, autoimmune hepatitis, and immune thrombocytopenic purpura, stratified by age group, sex, and race. Abbreviations: DAA, direct-acting antiviral; HR, hazard ratio; CI, confidence interval.
(DOCX)

## Abbreviations

HCV: hepatitis C virus
IMIDs: immune-mediated inflammatory diseases
DAA: direct-acting antiviral
SVR: sustained virologic response
ICD-10-CM: International Classification of Diseases, Tenth Revision, Clinical Modification
HBV: hepatitis B virus
HIV: human immunodeficiency virus
HRs: hazard ratios
CIs: confidence intervals
EBV: Epstein-Barr virus
SARS-CoV-2: severe acute respiratory syndrome coronavirus 2
MASLD: metabolic dysfunction-associated steatotic liver disease

## References

[1] Mazzaro C, Quartuccio L, Adinolfi LE, Roccatello D, Pozzato G, Nevola R, et al. A review on extrahepatic manifestations of chronic hepatitis C virus infection and the impact of direct-acting antiviral therapy. Viruses. 2021;13(11):2249. doi: 10.3390/v13112249 34835054 PMC8619859

[2] Ramos-Casals M, Muñoz S, Medina F, Jara L-J, Rosas J, Calvo-Alen J, et al. Systemic autoimmune diseases in patients with hepatitis C virus infection: characterization of 1020 cases (The HISPAMEC Registry). J Rheumatol. 2009;36(7):1442–8. doi: 10.3899/jrheum.080874 19369460

[3] McMurray RW, Elbourne K. Hepatitis C virus infection and autoimmunity. Semin Arthritis Rheum. 1997;26(4):689–701. doi: 10.1016/s0049-0172(97)80005-4 9062950

[4] Pastore F, Martocchia A, Stefanelli M, Prunas P, Giordano S, Toussan L, et al. Hepatitis C virus infection and thyroid autoimmune disorders: A model of interactions between the host and the environment. World J Hepatol. 2016;8(2):83–91. doi: 10.4254/wjh.v8.i2.83 26807204 PMC4716530

[5] Bertino G, Ardiri A, Proiti M, Rigano G, Frazzetto E, Demma S, et al. Chronic hepatitis C: This and the new era of treatment. World J Hepatol. 2016;8(2):92–106. doi: 10.4254/wjh.v8.i2.92 26807205 PMC4716531

[6] Dumoulin FL, Leifeld L, Sauerbruch T, Spengler U. Autoimmunity induced by interferon-alpha therapy for chronic viral hepatitis. Biomed Pharmacother. 1999;53(5–6):242–54. doi: 10.1016/S0753-3322(99)80095-X 10424246

[7] Shiffman ML. Side effects of medical therapy for chronic hepatitis C. Ann Hepatol. 2004;3(1):5–10. doi: 10.1016/s1665-2681(19)32118-0 15118573

[8] Zignego AL, Ramos-Casals M, Ferri C, Saadoun D, Arcaini L, Roccatello D, et al. International therapeutic guidelines for patients with HCV-related extrahepatic disorders. A multidisciplinary expert statement. Autoimmun Rev. 2017;16(5):523–41. doi: 10.1016/j.autrev.2017.03.004 28286108

[9] Kondili LA, Monti M, Quaranta MG, Gragnani L, Panetta V, Brancaccio G, et al. A prospective study of direct-acting antiviral effectiveness and relapse risk in HCV cryoglobulinemic vasculitis by the Italian PITER cohort. Hepatology. 2022;76(1):220–32. doi: 10.1002/hep.32281 34919289 PMC9305531

[10] Salama II, Raslan HM, Abdel-Latif GA, Salama SI, Sami SM, Shaaban FA, et al. Impact of direct-acting antiviral regimens on hepatic and extrahepatic manifestations of hepatitis C virus infection. World J Hepatol. 2022;14(6):1053–73. doi: 10.4254/wjh.v14.i6.1053 35978668 PMC9258264

[11] Ogawa E, Chien N, Kam L, Yeo YH, Ji F, Huang DQ, et al. Association of direct-acting antiviral therapy with liver and nonliver complications and long-term mortality in patients with chronic hepatitis C. JAMA Intern Med. 2023;183(2):97–105. doi: 10.1001/jamainternmed.2022.5699 36508196 PMC9856614

[12] Klein SL, Flanagan KL. Sex differences in immune responses. Nat Rev Immunol. 2016;16(10):626–38. doi: 10.1038/nri.2016.90 27546235

[13] Grebely J, Page K, Sacks-Davis R, van der Loeff MS, Rice TM, Bruneau J, et al. The effects of female sex, viral genotype, and IL28B genotype on spontaneous clearance of acute hepatitis C virus infection. Hepatology. 2014;59(1):109–20. doi: 10.1002/hep.26639 23908124 PMC3972017

[14] Mauvais-Jarvis F, Bairey Merz N, Barnes PJ, Brinton RD, Carrero J-J, DeMeo DL, et al. Sex and gender: modifiers of health, disease, and medicine. Lancet. 2020;396(10250):565–82. doi: 10.1016/S0140-6736(20)31561-0 32828189 PMC7440877

[15] Palchuk MB, London JW, Perez-Rey D, Drebert ZJ, Winer-Jones JP, Thompson CN, et al. A global federated real-world data and analytics platform for research. JAMIA Open. 2023;6(2):ooad035. doi: 10.1093/jamiaopen/ooad035 37193038 PMC10182857

[16] Kondo Y, Ninomiya M, Kimura O, Machida K, Funayama R, Nagashima T, et al. HCV infection enhances Th17 commitment, which could affect the pathogenesis of autoimmune diseases. PLoS One. 2014;9(6):e98521. doi: 10.1371/journal.pone.0098521 24905921 PMC4048196

[17] Sansonno L, Tucci FA, Sansonno S, Lauletta G, Troiani L, Sansonno D. B cells and HCV: an infection model of autoimmunity. Autoimmun Rev. 2009;9(2):93–4. doi: 10.1016/j.autrev.2009.03.008 19318140

[18] Wang Y, Dou H, Liu G, Yu L, Chen S, Min Y, et al. Hepatitis C virus infection and the risk of Sjögren or sicca syndrome: a meta-analysis. Microbiol Immunol. 2014;58(12):675–87. doi: 10.1111/1348-0421.12202 25263827

[19] Antonelli A, Ferri C, Pampana A, Fallahi P, Nesti C, Pasquini M, et al. Thyroid disorders in chronic hepatitis C. Am J Med. 2004;117(1):10–3. doi: 10.1016/j.amjmed.2004.01.023 15210382

[20] Jamalinia M, Lonardo A, Weiskirchen R. Sex and gender differences in liver fibrosis: pathomechanisms and clinical outcomes. Fibrosis. 2024;2(4):10006. doi: 10.70322/fibrosis.2024.10006

[21] Robinson WH, Younis S, Love ZZ, Steinman L, Lanz TV. Epstein-Barr virus as a potentiator of autoimmune diseases. Nat Rev Rheumatol. 2024;20(11):729–40. doi: 10.1038/s41584-024-01167-9 39390260

[22] Liu Y, Sawalha AH, Lu Q. COVID-19 and autoimmune diseases. Curr Opin Rheumatol. 2021;33(2):155–62. doi: 10.1097/BOR.0000000000000776 33332890 PMC7880581

[23] Bjornevik K, Cortese M, Healy BC, Kuhle J, Mina MJ, Leng Y, et al. Longitudinal analysis reveals high prevalence of Epstein-Barr virus associated with multiple sclerosis. Science. 2022;375(6578):296–301. doi: 10.1126/science.abj8222 35025605

[24] Inokuchi S, Shimamoto K. Persistent risk of developing autoimmune diseases associated with COVID-19: an observational study using an electronic medical record database in Japan. J Clin Rheumatol. 2024;30(2):65–72. doi: 10.1097/RHU.0000000000002054 38190730

[25] Meyer M, Schwärzler J, Jukic A, Tilg H. Innate Immunity and MASLD. Biomolecules. 2024;14(4):476. doi: 10.3390/biom14040476 38672492 PMC11048298

[26] Huang C-F, Dai C-Y, Lin Y-H, Wang C-W, Jang T-Y, Liang P-C, et al. Dynamic change of metabolic dysfunction-associated steatotic liver disease in chronic hepatitis C patients after viral eradication: a nationwide registry study in Taiwan. Clin Mol Hepatol. 2024;30(4):883–94. doi: 10.3350/cmh.2024.0414 39069721 PMC11540343

[27] Shengir M, Elgretli W, Cinque F, Ramanakumar AV, Lombardi R, Cespiati A, et al. Metabolic factors drive early increase in hepatic steatosis despite improvement in non-invasive fibrosis markers after hepatitis C eradication with direct-acting antivirals. Clin Res Hepatol Gastroenterol. 2025;49(7):102639. doi: 10.1016/j.clinre.2025.102639 40532848

