# Supplementary material for: Direct-acting antiviral therapy is associated with a reduced risk of selected immune-mediated inflammatory diseases in chronic hepatitis C infection: A real-world cohort study
Source: PLoS One. 2026 Jun 25;21(6):e0351973. doi: 10.1371/journal.pone.0351973 (PMC13298774; doi:10.1371/journal.pone.0351973)
Supplement: S1 Table — Comprehensive list of all ICD-10-CM codes used in the study, including definitions for immune-mediated inflammatory diseases, inclusion and exclusion criteria, covariates used for propensity score matching, and control outcomes. Abbreviations: HCV, hepatitis C virus; HBV, hepatitis B virus; HIV, human immunodeficiency virus. (DOCX) [file pone.0351973.s001.docx]

**S1 Table** ICD-10-CM code definitions.

| **Category** | **Condition/Variable** | **ICD-10-CM code(s)** |
| --- | --- | --- |
| IMID | Rheumatoid arthritis | M05-M06 |
|  | Systemic lupus erythematosus | M32 |
|  | Sjögren’s syndrome | M35.0 |
|  | Systemic sclerosis | M34 |
|  | Dermatopolymyositis | M33 |
|  | Sarcoidosis | D86 |
|  | Systemic vasculitis | M30-M31 |
|  | Antiphospholipid syndrome | D68.61 |
|  | Autoimmune hepatitis | K75.4 |
|  | Autoimmune thyroiditis | E06.3 |
|  | Immune thrombocytopenic purpura | D69.3 |
|  | Cutaneous vasculitis | L95 |
|  | Psoriasis | L40 |
| HCV infection | Chronic HCV infection | B18.2 |
| Exclusion criteria | HBV infection | B16, B17.0, B18.0, B18.1, B19.1, Z22.51 |
|  | HIV infection | B20, Z21 |
| Covariates (matching) | Problems related to housing and economic circumstances | Z59 |
|  | Employment and unemployment problems | Z56 |
|  | Education and literacy problems | Z55 |
|  | Occupational exposure to risk factors | Z57 |
|  | Tobacco use (proxy for smoking) | Z72.0 |
|  | Nicotine dependence (proxy for smoking) | F17 |
|  | Alcohol-related liver disease (proxy for alcohol use) | K70 |
|  | Hypertension | I10 |
|  | Type 2 diabetes mellitus | E11 |
|  | Hyperlipidemia | E78 |
|  | Chronic kidney disease | N18 |
|  | Asthma | J45 |
|  | Depression | F32 |
|  | Sleep disorder | G47 |
|  | Psychoactive substance use | F10-F19 |
|  | Vitamin D deficiency | E55 |
|  | Liver cirrhosis | K74.6 |
|  | Hepatic fibrosis | K74.0 |
|  | Neoplasms | C00-D49 |
| Positive control outcome | Liver cirrhosis | K74.0, K74.6 |
|  | Hepatocellular carcinoma | C22.0, C22.7, C22.8 |
|  | Cryoglobulinemia | D89.1 |
|  | Type 2 diabetes mellitus | E11 |
| Negative control outcome | Osteoarthritis | M15-M19 |
|  | Migraine | G43 |
|  | Acute appendicitis | K35 |

Abbreviations: IMID, immune-mediated inflammatory disease; HCV, hepatitis C virus; HBV, hepatitis B virus; HIV, human immunodeficiency virus.
