# Supplementary material for: Direct-acting antiviral therapy is associated with a reduced risk of selected immune-mediated inflammatory diseases in chronic hepatitis C infection: A real-world cohort study
Source: PLoS One. 2026 Jun 25;21(6):e0351973. doi: 10.1371/journal.pone.0351973 (PMC13298774; doi:10.1371/journal.pone.0351973)
Supplement: S2 Table — Hazard ratios for key immune-mediated inflammatory diseases (rheumatoid arthritis, autoimmune hepatitis, and immune thrombocytopenic purpura) under 60-day, 180-day (primary), and 365-day exclusion windows to assess robustness. Abbreviations: HR, hazard ratio; CI, confidence interval. (DOCX) [file pone.0351973.s002.docx]

**S2 Table** Sensitivity analyses using alternative post-index exclusion windows.

| **Outcomes** | **60-Day Window** | **180-Day Window (Main)** | **365-Day Window** |
| --- | --- | --- | --- |
|  | **HR (95% CI)** | **HR (95% CI)** | **HR (95% CI)** |
| **Systemic IMID** |  |  |  |
| Rheumatoid arthritis | **0.84 (0.72–0.97)** | **0.83 (0.71–0.97)** | **0.83 (0.70–0.98)** |
| Systemic lupus erythematosus | 0.78 (0.55–1.11) | 0.81 (0.55–1.19) | 0.83 (0.55–1.27) |
| Sjögren's syndrome | 1.26 (0.89–1.80) | 1.29 (0.90–1.86) | 1.39 (0.92–2.10) |
| Systemic sclerosis | 1.36 (0.57–3.24) | 1.54 (0.63–3.76) | 1.16 (0.45–3.01) |
| Dermatopolymyositis | 0.62 (0.24–1.58) | 0.65 (0.24–1.79) | 0.73 (0.26–2.04) |
| Sarcoidosis | 1.10 (0.75–1.62) | 1.13 (0.74–1.74) | 1.04 (0.66–1.63) |
| Systemic vasculitis | **0.73 (0.55–0.98)** | 0.74 (0.55–1.01) | 0.82 (0.59–1.14) |
| Antiphospholipid syndrome | 0.95 (0.54–1.67) | 1.14 (0.62–2.09) | 1.24 (0.64–2.42) |
| **Organ-specific IMID** |  |  |  |
| Autoimmune hepatitis | 0.61 (0.37–1.01) | **0.55 (0.31–0.96)** | **0.41 (0.21–0.80)** |
| Autoimmune thyroiditis | 1.20 (0.89–1.62) | 1.20 (0.88–1.65) | 1.16 (0.83–1.62) |
| Immune thrombocytopenic purpura | **0.65 (0.45–0.92)** | **0.64 (0.44–0.93)** | **0.65 (0.43–1.00)** |
| Cutaneous vasculitis | 0.71 (0.49–1.04) | 0.77 (0.51–1.15) | 0.71 (0.45–1.11) |
| Psoriasis | 1.13 (0.95–1.34) | 1.14 (0.95–1.37) | 1.14 (0.94–1.39) |
| **Positive control outcome** |  |  |  |
| Liver cirrhosis | **0.73 (0.70–0.77)** | **0.72 (0.68–0.76)** | **0.64 (0.59–0.68)** |
| Hepatocellular carcinoma | **0.78 (0.71–0.86)** | **0.88 (0.80–0.97)** | 0.92 (0.82–1.02) |
| Cryoglobulinemia | **0.70 (0.51–0.96)** | **0.66 (0.45–0.95)** | 0.65 (0.42–1.01) |
| Type 2 diabetes mellitus | **0.77 (0.72–0.82)** | **0.79 (0.74–0.85)** | **0.83 (0.77–0.89)** |
| **Negative control outcome** |  |  |  |
| Osteoarthritis | 1.01 (0.97–1.04) | 1.01 (0.98–1.04) | 1.03 (0.99–1.06) |
| Migraine | 1.04 (0.97–1.12) | 1.06 (0.99–1.14) | 1.08 (0.99–1.16) |
| Acute appendicitis | 0.94 (0.71–1.25) | 0.94 (0.70–1.26) | 0.85 (0.62–1.19) |

Bold font indicates statistically significant results (*P* < 0.05).

Abbreviations: HR, hazard ratio; CI, confidence interval; IMID, immune-mediated inflammatory disease.
