# Supplementary material for: Direct-acting antiviral therapy is associated with a reduced risk of selected immune-mediated inflammatory diseases in chronic hepatitis C infection: A real-world cohort study
Source: PLoS One. 2026 Jun 25;21(6):e0351973. doi: 10.1371/journal.pone.0351973 (PMC13298774; doi:10.1371/journal.pone.0351973)
Supplement: S3 Table — Hazard ratios and 95% confidence intervals for rheumatoid arthritis, autoimmune hepatitis, and immune thrombocytopenic purpura, stratified by age group, sex, and race. Abbreviations: DAA, direct-acting antiviral; HR, hazard ratio; CI, confidence interval. (DOCX) [file pone.0351973.s003.docx]

**S3 Table** Stratified analysis of immune-mediated inflammatory disease risk by demographic subgroups.

| **Outcome** | **Subgroup** | **DAA-treated** | **Untreated** | **HR (95% CI)** |
| --- | --- | --- | --- | --- |
|  |  | **Events** | **Events** |  |
| Rheumatoid arthritis |  |  |  |  |
|  | Male | 124 | 147 | 0.87 (0.68–1.10) |
|  | Female | 159 | 211 | **0.80 (0.65–0.98)** |
|  | White | 198 | 225 | 0.91 (0.75–1.10) |
|  | Black | 66 | 103 | **0.66** (**0.48–0.90)** |
|  | Latino | 18 | 14 | 1.44 (0.72–2.91) |
|  | 18-40y | 17 | 17 | 1.24 (0.63–2.43) |
|  | 41-64y | 101 | 156 | **0.69 (0.54–0.89)** |
|  | ≥ 65y | 148 | 170 | **0.80 (0.64–0.99)** |
| Autoimmune hepatitis |  |  |  |  |
|  | Male | ≤10† | 13 | 0.76 (0.33–1.73) |
|  | Female | ≤10† | 17 | 0.43 (0.18–1.04) |
|  | White | 13 | 26 | **0.51 (0.26–0.98)** |
|  | Black | ≤10† | ≤10† | 1.24 (0.33–4.60) |
|  | Latino | ≤10† | ≤10† | 0.35 (0.04–3.35) |
|  | 18-40y | ≤10† | ≤10† | 1.07 (0.15–7.59) |
|  | 41-64y | ≤10† | ≤10† | 1.19 (0.43–3.28) |
|  | ≥ 65y | ≤10† | 25 | **0.25 (0.11–0.59)** |
| Immune thrombocytopenic purpura |  |  |  |  |
|  | Male | 29 | 45 | 0.66 (0.41–1.05) |
|  | Female | 14 | 28 | 0.53 (0.28–1.01) |
|  | White | 29 | 49 | **0.58 (0.37–0.93)** |
|  | Black | 11 | 14 | 0.82 (0.37–1.80) |
|  | Latino | ≤10† | ≤10† | 0.66 (0.16–2.75) |
|  | 18-40y | ≤10† | ≤10† | 0.89 (0.20–4.00) |
|  | 41-64y | ≤10† | 33 | **0.26 (0.12–0.57)** |
|  | ≥ 65y | 29 | 30 | 0.88 (0.53–1.47) |

†To protect patient privacy, numbers are rounded up to 10.

Bold font indicates statistically significant results (*P* < 0.05).

Abbreviations: DAA, direct-acting antiviral; HR, hazard ratio; CI, confidence interval.
